# Causal relationship between dietary salt intake and dementia risk: Mendelian randomization study

**DOI:** 10.1186/s12263-024-00741-w

**Published:** 2024-03-15

**Authors:** Ke Shi, Yongbo Yu, Zhaolin Li, Miaomiao Hou, Xinyi Li

**Affiliations:** Third Hospital of Shanxi Medical University, Shanxi Bethune Hospital, Tongji Shanxi Hospital, Shanxi Academy of Medical Sciences, Taiyuan, 030032 China

**Keywords:** Dietary salt intake, Dementia, Mendelian randomization, Causal association, Genetic instrument, Single-nucleotide polymorphisms

## Abstract

**Objective:**

Observational research has indicated a potential link between dietary salt intake and susceptibility to dementia. However, it is important to note that these types of studies are prone to the issues of reverse causation and residual confounding. Therefore, we conducted a two-sample Mendelian randomization (MR) study to explore the causality.

**Method:**

To explore the causal relationship between them, this Mendelian randomization (MR) study incorporated summary statistics of dietary salt intake and dementia. We estimated the causality between salt intake and the risk of overall dementia and various subtypes of dementia, including Alzheimer’s disease (AD), Vascular dementia (VaD), and Lewy body dementia (LBD). The inverse variance-weighted (IVW) method was the major MR analysis. To conduct sensitivity analyses, we employed various MR methods, the pleiotropy residual sum and outlier (MR-PRESSO) method, and the leave-one-out approach. The MR-Egger intercept and Cochran’s Q test were conducted to test pleiotropy and heterogeneity respectively.

**Results:**

A suggestive association was observed for genetically predicted higher dietary salt intake and increased risk of overall dementia in the European ancestry [odds ratio (OR): 1.542; 95% confidence interval (95% CI): 1.095–2.169;* P* = 0.013]. The causal relationship between dietary salt intake and overall dementia is robust with respect to the choice of statistical methods and is validated through extensive sensitivity analyses that guard against various model assumption violations. Meanwhile, no clear heterogeneity or pleiotropy was identified. However, we failed to detect a causal effect of dietary salt intake on the risk of various dementia subtypes.

**Conclusion:**

The results of this research present strong evidence that established a significant association between dietary salt intake and the likelihood of developing dementia. These findings reinforce the notion that the amount of dietary salt intake plays a crucial role in determining the risk of acquiring this cognitive condition. By establishing a definitive correlation, this study highlights the importance of reducing salt consumption as a preventive measure against dementia.

**Supplementary Information:**

The online version contains supplementary material available at 10.1186/s12263-024-00741-w.

## Introduction

Dementia is a destructive neurodegenerative disorder that presents with memory loss, cognitive decline, and mental disabilities [1, 2], significantly impacting the daily lives of half a million individuals [3]. Unfortunately, there are currently no curative treatments or medications that can effectively prevent dementia [4, 5]. Hence, it is imperative to conduct investigations to recognize and examine precautionary approaches in order to diminish or avert the initiation of cognitive decay in vulnerable individuals and lower the occurrence of progression into clinical dementia [6–8].

In recent times, there has been an increasing focus on the connection between dietary patterns and well-being [9–11]. Observational studies have suggested that dietary components may influence the pathological process of dementia. Several studies have found an epidemiological association between high salt intake and memory loss as well as an increased risk of dementia [12, 13]. For example, a recent large-scale prospective observational study reported that excessive salt intake exacerbated the progression of cognitive impairment and increased dementia risk [13], and another population-based cohort study demonstrated similar findings [12]. However, there are conflicting results from other studies that have not found a significant link between salt intake and dementia risk [14]. These inconsistent findings may be attributed to variations in study populations, small sample sizes, or the influence of other confounding factors. Due to these confounding factors, it is challenging for observational studies to determine the independent effects of salt intake on risk of dementia.

Mendelian randomization (MR) analysis utilizes genetic variation as an instrumental variable to establish causal relationships between exposures and outcomes [15, 16]. By using a two-sample MR design, the limitations of traditional epidemiological studies can be partially overcome [17]. Due to the natural and random distribution of genetic variants, MR is less prone to confounding and reverse causation [18]. The existing literature primarily examines the overall influence of salt consumption on cognitive function and dementia, with limited research conducted on the correlation between salt intake and dementia. In our study, we attempted to investigate a potential causal relationship between salt intake and dementia risk using Mendelian randomization methods.

## Methods

### Study design

**Fig. 1:**
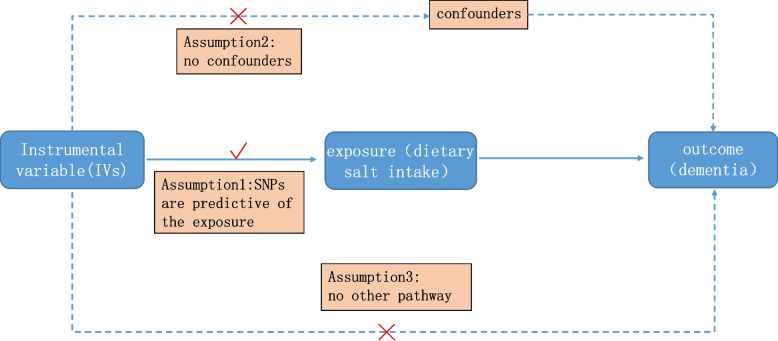
Design flow chart in the MR study. MR assumptions: assumption 1, 2, and 3. Solid line represents direct causal effects that genetic instrument variants are reliably associated with dietary salt intake levels and influence the dementia risk through the dietary salt intake in assumption 1. The dotted line represents that dietary salt genetic instrument variants are not associated with any measured or unmeasured confounders and do not influence the risk of dementia through other pathways in assumptions 2 and 3, respectively

This study utilized a two-sample MR design and incorporated summary-level data on dietary salt intake and various dementia subtypes from independent nonoverlapping populations. Three assumptions, as depicted in Fig. 1, were considered essential for MR studies: (I) significant association between genetic variants and dietary salt intake (*P* < 5 × 10^−8^), (II) the absence of genetic variants’ association with any confounding factors, and (III) genetic variants solely linked to dementia through dietary salt intake [19, 20]. The study design is summarized in Fig. 1. Ethical consent from participants was not required as this MR study relied on publicly available databases.

### Data sources

Summary-level data on dietary salt intake were obtained from the UK Biobank database including up to 462,630 participants of European ancestry. All dietary data were evaluated using questionnaires as categorical variables. The inquiry regarding dietary salt intake entailed the following query: “Do you add salt to your food? (Do not include salt used in cooking).” The available choices for response were as follows: “never/rarely,” “usually,” “sometimes,” “always,” and “prefer not to answer” (https://biobank.ctsu.ox.ac.uk/crystal/field.cgi?id=1478).

The FinnGen study is a global research project that aims to collect and analyze the genome and health data of half a million Finns [21]. The summary-level data for Alzheimer’s disease (AD) were obtained from the FinnGen consortium, including 3899 AD cases and 214,893 controls of European descent. In the case of Vascular dementia (VaD), the GWAS summary statistics used in this study were obtained from the FinnGen consortium, and it consisted of 387 cases and 211,300 normal controls of European ancestry. As for Lewy body dementia (LBD), the GWAS summary statistics were obtained from a recent study conducted by Chia et al., including a total of 2591 cases and 4027 controls of European ancestry. The GWAS summary statistics of overall dementia were from the FinnGen consortium, including 7284 cases and 209,487 normal controls of European ancestry.

Table 1 provides a comprehensive overview of the specifics encompassing the datasets used in the current MR study. All the GWAS summary data within the analyses of the present MR study were obtained from the IEU OpenGWAS project (IEU OpenGWAS project (mrcieu.ac.uk)).

**Table 1:** Details of studies included in Mendelian randomization analyses

Exposure/outcome	GWAS ID	Consortium/author	Sample size	SNPs number	Ethnicity	Year
Dietary salt intake	ukb-b-8,121	UKB	462,630	9,851,867	European	2018
AD	finn-b-G6_ALZHEIMER	FinnGen project	218,792	16,380,466	European	2021
VaD	Finn-b-VD_U	FinnGen project	211,687	16,380,454	European	2021
LBD	ebi-a-GCST90001390	Chia R	6618	7,593,175	European	2021
Dementia	Finn-b-F5_DEMENTIA	FinnGen project	216,771	16,380,463	European	2021

*Abbreviations*: *AD* Alzheimer’s disease, *GWAS* genome-wide association study, *LBD* Lewy body dementia, *SNP* single-nucleotide polymorphism, *UKB* United Kingdom Biobank, *VaD* vascular dementia

### Selection of genetic instruments

We extracted single-nucleotide polymorphisms (SNPs) from GWAS data based on two strong correlation and independence criteria: genome-wide statistical significance level (5 × 10^−8^) and linkage disequilibrium (LD) and *r*^2^ < 0.001 and clustering windows > 10,000 kb. Furthermore, all SNPs were cross-referenced with the PhenoScanner database V2 (http://www.phenoscanner.medschl.cam.ac.uk/) to verify associations with confounders and outcomes [22]. F-statistics were calculated to assess the strength of genetic variants [23].

### Statistical analysis

We used the inverse variance-weighted (IVW) method as the primary analysis to evaluate the relationship between dietary salt intake and dementia by combining the *β*-values and the standard errors of the causal estimate from them [15, 24]. Whenever no significant heterogeneity was detected by the Cochran Q test (*P* > 0.05), a fixed-effect model was implemented; otherwise, a random-effects model was used [15]. The results were presented as odds ratios (ORs) and 95% confidence intervals (CIs) for the association between dietary salt intake and dementia. Sensitivity analyses were further performed by using multiple MR methods, the pleiotropy residual sum and outlier (MR-PRESSO) method, and the leave-one-out approach. The MR-PRESSO method was employed to detect outliers, which were promptly removed [25]. After removing outliers, the MR analysis was repeated. The leave-one-out analysis was conducted to evaluate the impact of removing a single SNP on the results [26, 27]. The MR-Egger intercept and Cochran’s Q test were conducted to test pleiotropy and heterogeneity. The strength of each SNP was assessed by F-statistic using the formula *F* = R2 (N-2)/(1-R2), where *R*^2^ was the proportion of total variation in the exposure that is explained by the genetic instruments and N was the total sample size [28, 29]. The statistical software R (version 4.2.2, R Foundation for Statistical Computing, Vienna, Austria; https://www.R-project.org) was utilized to carry out all statistical analyses and visualization. The packages employed for this purpose were “Two Sample MR,” “LDlinkR,” and “forest plot” [30]. Lastly, we calculated the statistical power of our MR analyses using the online calculator mRnd (https://shiny.cnsgenomics.com/mRnd/) [31].

## Results

Initially, we identified 106 SNPs associated with dietary salt intake at the genome-wide significance level (*P* < 5 × 10^−8^), as shown in Supplementary Table 1. Based on the PhenoScanner database V2, 22 genetic instrumental variables directly related to confounding factors (including age, body mass index, and level of education) were removed (Supplementary Table 2). We further deleted SNPs with palindromic or incompatible alleles (rs55897719, rs13084934, rs6443950, rs9375448). Finally, these strictly selected SNPs were used as instrumental variables in the subsequent MR analysis. The F-statistics of these SNPs were all above the threshold of 10 (range, 29.742–224.897) (Supplementary Table 3).

**Fig. 2:**
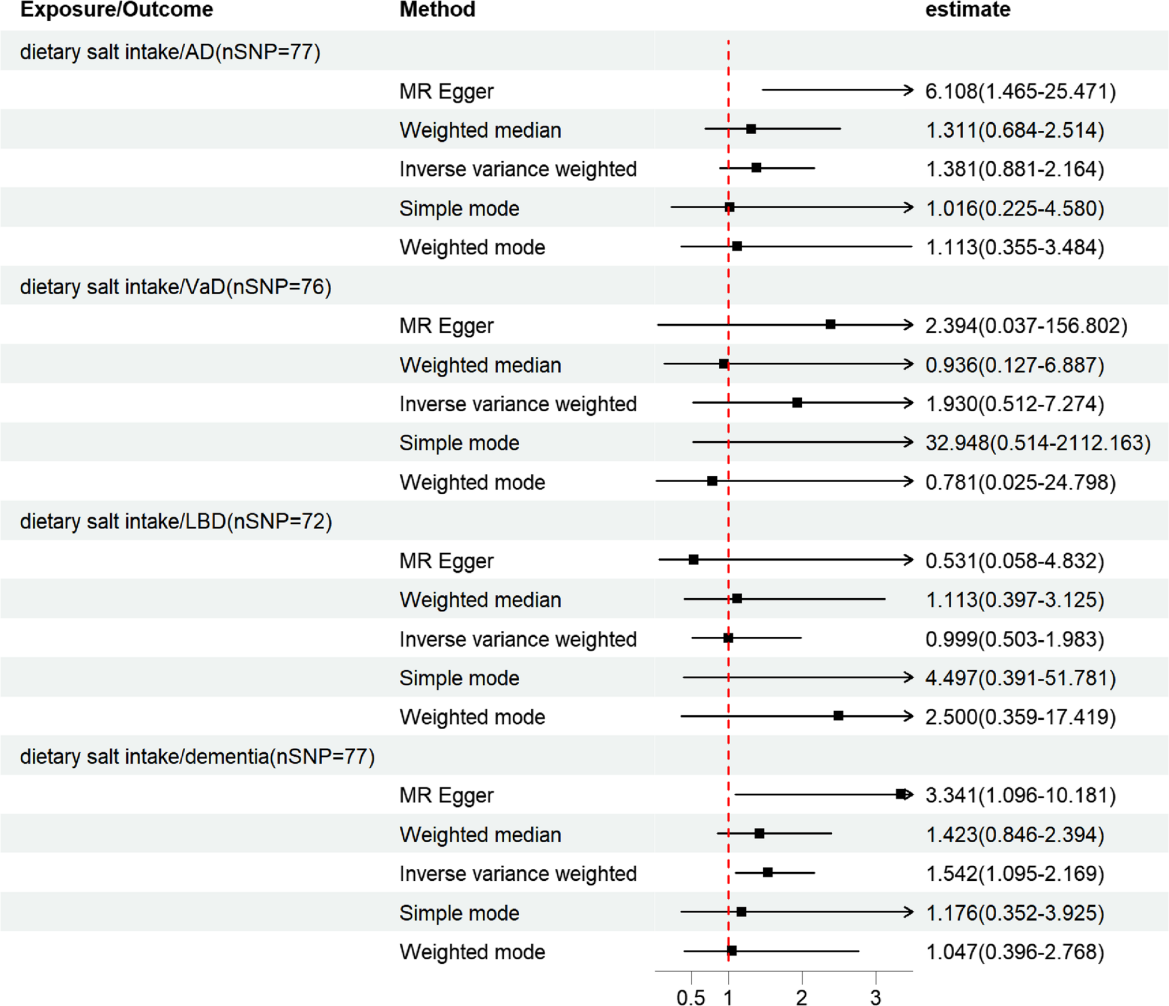
Forest plot in the MR study. Forest plot showing results from the Mendelian randomization study to evaluate potential causal associations between dietary salt intake and risk of dementia. Abbreviations: AD, Alzheimer’s disease; LBD, Lewy body dementia; MR-Egger, Mendelian randomization-Egger; SNPs, single-nucleotide polymorphisms; VaD, vascular dementia

**Fig. 3:**
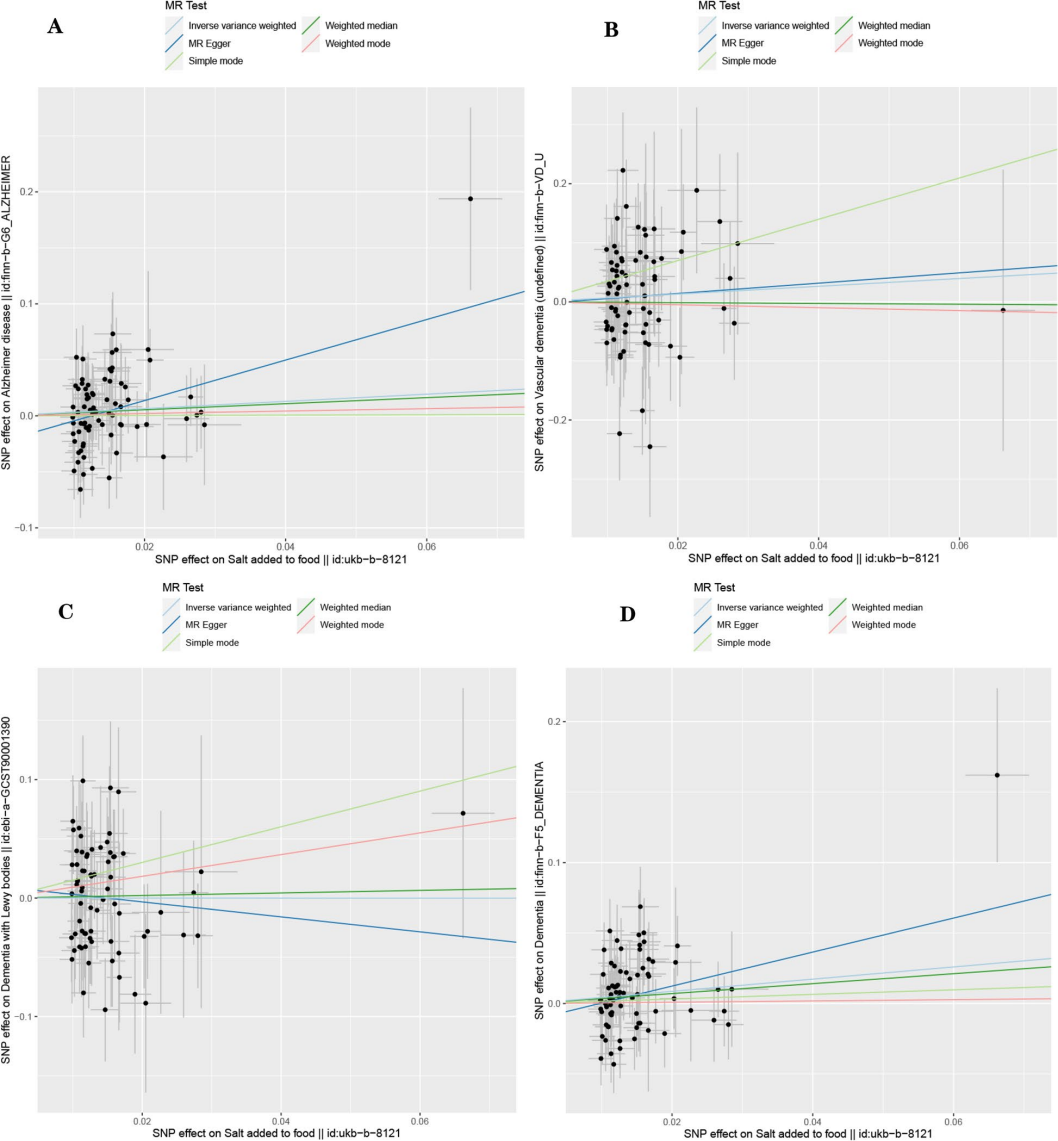
Scatter plot in the MR study. Scatter plot for two-sample MR analysis of causal relationship between dietary salt intake and risk of dementia using five MR methods: **A** Alzheimer’s disease, **B** Vascular dementia, **C** Lewy body dementia, **D** dementia. The *β*-value with SE is plotted to demonstrate effect estimate of each SNP for causal association of dietary salt intake (*x*-axis) with dementia (*y*-axis). The slope of each line represents the two-sample MR estimate (*β*-value) for the individual SNP. Error bar represents SE of effect size. Abbreviations: MR, Mendelian randomization; SE, standard error; SNP, number of single-nucleotide polymorphism

**Fig. 4:**
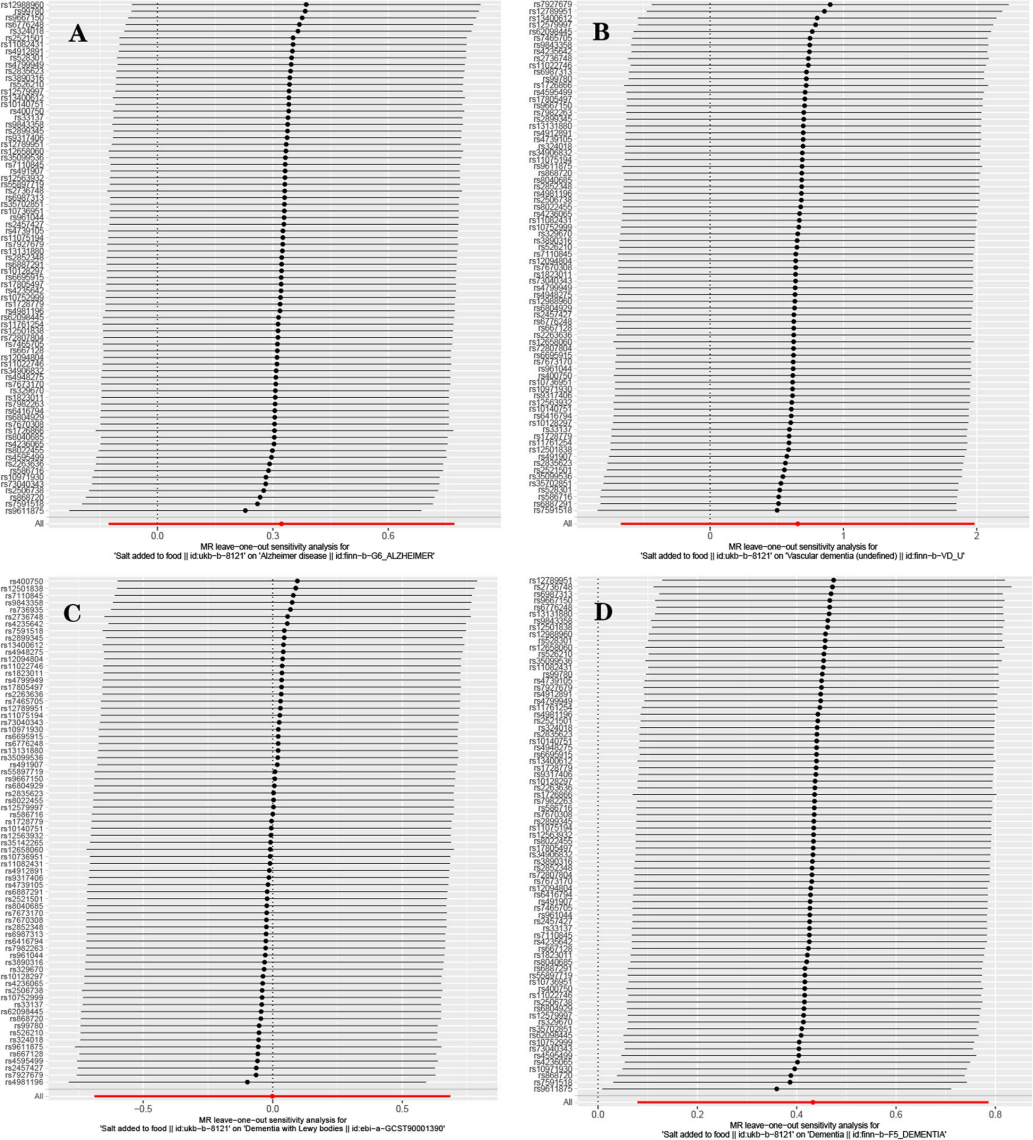
Leave-one-out plot in the MR study. Leave-one-out plot for sensitivity analysis of single SNP effect on dietary salt intake to dementia results: **A** Alzheimer’s disease, **B** Vascular dementia, **C** Lewy body dementia, **D** dementia. Leave-one-out plot using IVW method by sequentially re-evaluating the causal estimate after discarding one SNP at a time, which helps determine whether the overall effect is driven by the specific genetic variant. The black point denotes the causal effect estimate of dietary salt intake on dementia after discarding a certain SNP, and the black line signifies the 95% CI of estimate. The red point symbolizes the causal effect estimate of dietary salt intake on dementia with the valid SNPs, and the red line indicates the 95% CI of the estimate. Abbreviations: CI, confidence interval; SNP, number of single-nucleotide polymorphism

**Fig. 5:**
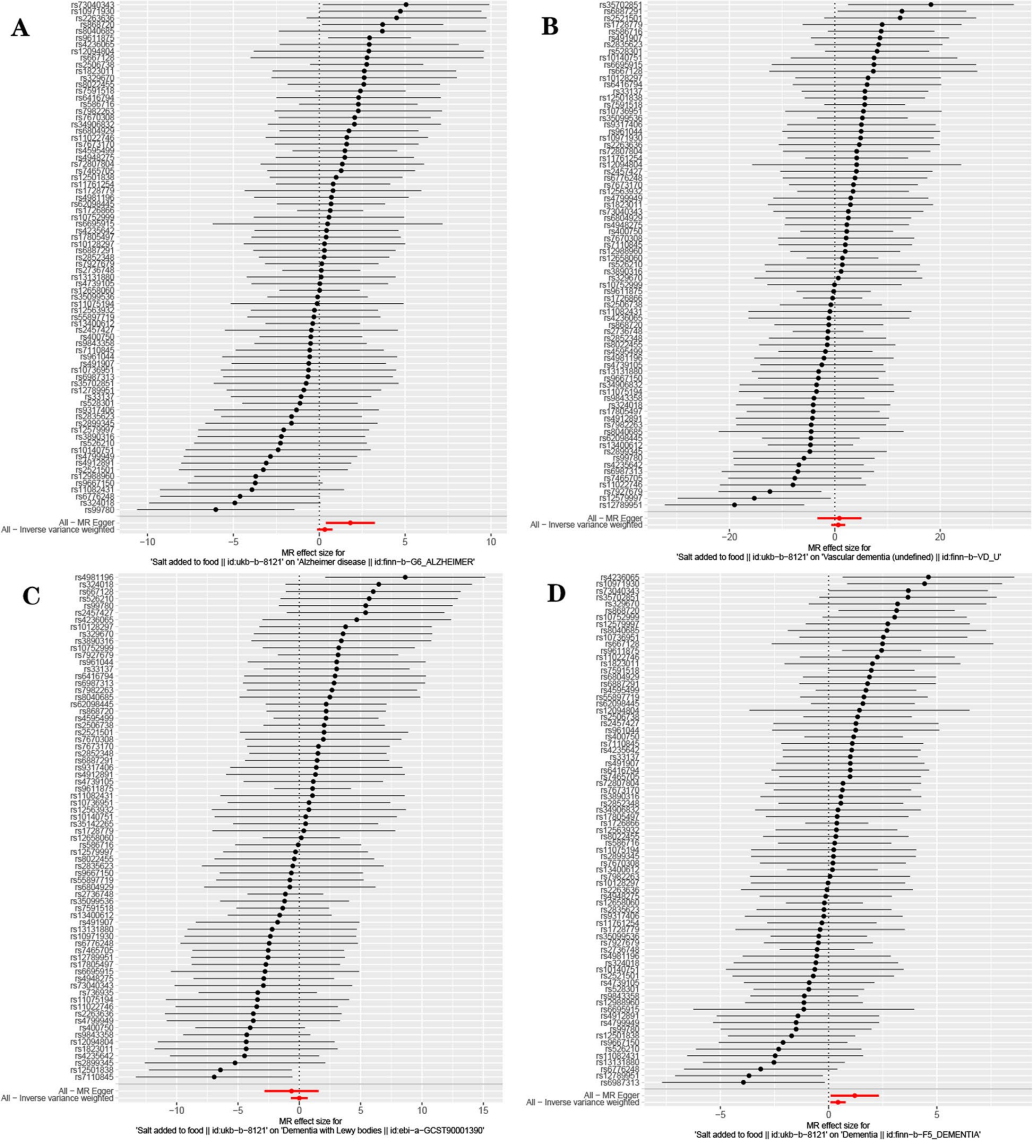
Forest plot in the MR study. Forest plot for two-sample MR analysis of single and summarized SNPs effects on relationship between dietary salt intake and risk of dementia with SNPs: **A** Alzheimer’ s disease, **B** Vascular dementia, **C** Lewy body dementia, **D** dementia. A black point denotes the effect estimate of dietary salt intake on dementia using a single SNP, and the black line signifies the 95% CI of the estimate. The red point symbolizes overall effect estimate of dietary salt intake and dementia with SNPs using the Egger and IVW method, and the red line indicates the 95% CI of the estimate. Abbreviations: CI, confidence interval; SNP, number of single-nucleotide polymorphism

Based on 77 SNPs associated with salt intake, we found a causal effect of salt intake on risk of overall dementia in our MR analysis (IVW: *OR* = 1.542, 95% *CI*: 1.095–2.169, *P* = 0.013; MR-Egger: *OR* = 3.341, 95% *CI*: 1.096–10.181, *P* = 0.037; weighted median: *OR* = 1.423, 95% *CI*: 0.846–2.394, *P* = 0.183; simple mode: *OR* = 1.176, 95% *CI*: 0.352–3.925, *P* = 0.793; weighted mode: *OR* = 1.047, 95% *CI*: 0.396–2.768, *P* = 0.927) (Fig. 2). The *P*-value of Cochran’s Q test indicated the absence of heterogeneity (MR-Egger: Q statistic = 79.219, *P* = 0.347; IVW: Q statistic = 81.389, *P* = 0.315). Moreover, the MR-Egger intercept test indicated the absence of pleiotropy (*P* = 0.156 [MR-Egger intercept test]). MR-PRESSO detected no outliers (*P* for global test of pleiotropy = 0.310), and the raw estimate is presented. Scatterplot shows a linear regression line for the positive association between salt intake and risk of overall dementia (Fig. 3D). The leave-one-out sensitivity analysis showed no single SNP has a substantial impact on the results, and the causality of genetically predicted salt intake on overall dementia was robust (Fig. 4D). The forest plot demonstrates the summary estimates of causal relationships between salt intake and risk of overall dementia (Fig. 5D). We estimated the statistical power to detect an OR of 0.99 in the risk of overall dementia per unit change of dietary salt intake.

However, our research did not find a correlation between salt intake and AD (IVW: *OR* = 1.381, 95% *CI*: 0.881–2.164, *P* = 0.160), VaD (IVW: *OR* = 1.930, 95% *CI*: 0.512–7.274, *P* = 0.331), LBD (IVW: *OR* = 0.999, 95% *CI*: 0.503–1.983, *P* = 0.997) (Fig. 2). There was no evidence that pleiotropy, heterogeneity, or outliers had biased these results (Table 2).

**Table 2:** Heterogeneity and pleiotropy estimates for the associations between dietary salt intake and dementia

Exposures/outcome	Heterogeneity test (MR-Egger)		Pleiotropy test			MR-PRESSO		
	Q	*p*-value	Egger intercept	SE	*p*-value	Causal estimate	SD	*p*-value
Dietary salt intake/AD	71.262	0.601	− 0.023	0.010	0.035	0.322	0.229	0.164
Dietary salt intake/VaD	67.110	0.702	− 0.003	0.031	0.916	0.658	0.636	0.304
Dietary salt intake/LBD	70.986	0.445	0.009	0.016	0.557	− 0.001	0.351	0.997
Dietary salt intake/dementia	79.219	0.347	− 0.012	0.008	0.156	0.433	0.180	0.019

*Abbreviations*: *AD* Alzheimer’s disease, *LBD* Lewy body dementia, MR-Egger Mendelian randomization-Egger, *MR-PRESSO* pleiotropy residual sum and outlier, *SE* standard error, *VaD* vascular dementia

## Discussion

Our MR study aimed to examine the causality between dietary salt intake and risks of dementia. To the best of our knowledge, this study represents the first analysis of the causal relationship between dietary salt intake and the risk of dementia, including various subtypes of dementia. Our findings demonstrated that higher dietary salt intake is associated with increased risk of overall dementia. The F-statistic for each SNP was above the threshold of 10, indicating that the selected SNPs are robust instruments of dietary salt intake. Multiple sensitivity analyses confirmed the robustness of our study findings.

In the last few years, several studies have linked dietary salt intake with dementia in the general population [12–14, 32]. In 2016, a prospective study investigated cognitively intact women participating in the Health Initiatives Memory Study (WHIMS) followed for 65.79 years (median follow-up period) and found that sodium intake > 1500 mg/day tended to increase the risk of MCI/PD in women with hypertension (*HR* = 1.2495% *CI*: 1.02–1.52) and taking antihypertensive drugs (*HR* = 1.1995% *CI*: 0.97–1.46) [13]. Similarly, a Chinese comprehensive analysis revealed that in the subset of high-quality studies, 75% of them reported a positive correlation between increased sodium intake and cognitive function [32]. A recent study in China indicated that excessive dietary salt increases the risk of dementia among the elderly who are independent of known risk factors [12]. Similarly, the findings of our MR study also demonstrate that high salt intake increases the risk of overall dementia. However, another ongoing multigenerational cohort study showed no significant association between sodium intake and dementia risk (*OR* = 1.64, 95% *CI*: 0.95–2.83; *n* = 2461) [14]. Almost all observational studies used cross-sectional designs and therefore have inherent limitations such as unknown confounding factors and reverse causal association. It is worthwhile pointing out the causality of dietary salt intake and risk of dementia by unconventional approaches (particularly MR study) to avoid confounding.

In addition, there is no evidence that suggests a causal association of dietary salt intake with other dementia subtypes (including AD, VaD, and LBD) in the present MR studies. This discrepancy may suggest the existence of correlation rather than causality between dietary salt intake and other dementia subtypes, which warrants further research to elucidate the underlying relationship.

The underlying mechanisms of the correlation between salt intake and dementia, however, are still elusive. Previous findings indicate that hypertensive rats fed a high salt diet exhibit cognitive impairment accompanied by abnormalities in synaptic plasticity [33, 34]. It is well-known that the hyperphosphorylation of the microtubule-associated protein tau is widely recognized as a contributor to neuronal loss and cognitive impairment in AD and dementia [35]. A recent study has provided evidence that tau hyperphosphorylation plays a role in mediating cognitive impairment induced by a high-salt diet [36]. Several studies in rodents have found adverse cognitive effects of high salt intake, with impairments often linked to oxidative stress markers [33, 37, 38]. Taken together, dietary salt intake may play a critical role in the pathogenesis of dementia.

Our study has several strengths. Firstly, we have presented additional proof to reinforce the causal association connecting dietary salt intake and the potential for developing dementia. Moreover, by considering the random distribution of genetic variants within the population, we have minimized the potential for reverse causality and residual bias. It is important to note that all the data analyzed in our study were exclusively derived from individuals of European descent, thus mitigating any bias resulting from population stratification.

However, it is important to acknowledge the limitations of our study. As there are currently no GWAS studies on 24-h urinary sodium, we had to rely on the frequency of salt added to food as a proxy for estimating daily salt intake. While this is a common approach, it is not the gold standard method for measuring salt intake. Secondly, the frequency of added salt to food was self-reported by participants in the UK Biobank, which introduces the possibility of report bias. Thirdly, using the frequency of added salt in food as an exposure variable does not allow for a quantitative assessment of the relationship between salt intake and risk of dementia.

## Conclusion

In conclusion, the current MR study suggests that genetically determined higher dietary salt intake is significantly associated with an increased risk of dementia. Future studies will need to further clarify this relationship and confirm the generality of our results to socioeconomically and ethnically diverse populations.

### Supplementary Information

**Supplementary Material 1.**

## Data Availability

All GWAS summary statistics used in this study are publicly available. The summary statistics for the genetic associations of dietary salt intake (GWAS ID: ukb-b-8121), Alzheimer’s disease (GWAS ID: finn-b-G6_ALZHEIMER), vascular dementia (GWAS ID: Finn-b-VD_U), Lewy body dementia (GWAS ID: ebi-a-GCST90001390), and dementia GWAS datasets (GWAS ID: Finn-b-F5_DEMENTIA) can be found on the IEU OpenGWAS project at https://gwas.mrcieu.ac.uk/.

## Abbreviations

AD: Alzheimer’s disease
GWAS: Genome-wide association study
IVW: Inverse-variance weighted
LD: Linkage disequilibrium
LBD: Lewy body dementia
MR: Mendelian randomization
MR-Egger: Mendelian randomization-Egger
MR-PRESSO: MR pleiotropy residual sum and outlier
VaD: Vascular dementia
